# Age-Related Differences in Speech Production and Resting State Functional Network Dynamics

**DOI:** 10.1162/NOL.a.208

**Published:** 2026-01-13

**Authors:** Haoyun Zhang, Keikei Lei, Hanxiang Yu, Megan Nakamura, Michele Diaz

**Affiliations:** Centre for Cognitive and Brain Sciences, University of Macau, Taipa, Macau SAR, China; Department of Psychology, University of Macau, Taipa, Macau SAR, China; Department of Psychology, Pennsylvania State University, University Park, PA, USA

**Keywords:** aging, ALFF, brain networks, functional connectivity, resting state fMRI, speech production

## Abstract

Age-related declines in cognitive function are often accompanied by changes in brain activity and network organization. This study investigated the relationship between resting state brain activity and age-related differences in speech production. We hypothesized that older adults would exhibit altered functional connectivity and activation intensity, correlating with reduced speech quality. Resting state functional MRI data were collected and a composite measure of speech complexity and fluency was calculated from younger and older adults. Results revealed significantly worse speech performance in older adults, accompanied by less segregated whole-brain networks, reduced amplitude of low-frequency fluctuations, and more heterogeneous brain states. Univariate regression analyses indicated stronger brain-behavior relationships in younger adults, while multivariate regression analyses revealed that age-related differences in resting state brain state patterns critically relate to speech production differences. Notably, the language network remained relatively stable with age, whereas whole-brain status became very important for speech performance in older adults. These findings suggest that resting state brain activity, particularly whole brain network characteristics, may serve as a stable biomarker of age-related changes in speech production.

## INTRODUCTION

As people age, various cognitive functions decline, including processing speed, working memory (Park, 2002; Park & Reuter-Lorenz, 2009), cognitive control (Paxton et al., 2008), and language production (Burke & Shafto, 2008). In speech production specifically, older adults often exhibit declines characterized by simpler and more disfluent speech (Horton et al., 2010; Kemper et al., 1989; Moscoso del Prado Martín, 2017). Along with these behavioral changes, aging is also associated with structural and functional brain decline, such as reduced gray matter volume (Sowell et al., 2003, 2004; Winkler et al., 2003) and dedifferentiated, that is, less segregated brain networks (Zhang, Gertel, et al., 2021). In particular, resting state functional activation data, which reflect how the brain operates in a task-free state, has been particularly useful in understanding age-related cognitive differences. Despite well-documented age-related differences in speech production, little research has examined how task-free brain states relate to speech performance. This study addresses this gap by investigating this relationship, integrating a comprehensive measure of speech quality, and modeling resting state brain activity using multiple methodological approaches.

Speech is a fundamental aspect of human communication, involving various processes such as language formulation, motor coordination, and other cognitive functions (Dien et al., 2008; Heald & Nusbaum, 2014; Kent, 2000). With aging, speech production undergoes notable changes, including slower speech rates (Diaz et al., 2016; Duchin & Mysak, 1987), increased disfluency (Bortfeld et al., 2001; Horton et al., 2010; Obler & Albert, 1981), reduced grammatical complexity (e.g., Kemper et al., 1989), and a higher tendency for off-topic speech (James et al., 1998). Reduced fluency and lower syntactic complexity often co-occur in individuals with weaker speech production abilities (Horton et al., 2010; Kemper et al., 2003, 2009). In fact, measures capturing different dimensions of speech are typically interrelated. For example, lexical and syntactic complexity have been shown to correlate with the proportion of disfluencies in connected speech (Fichman et al., 2024; Nippold, 2019). This pattern of interdependence supports the use of a composite score to capture meaningful individual differences in overall speech production ability. Using integrated speech measures, a previous study found that older adults produce lower-quality speech compared to younger adults, reflected by reduced syntactic complexity and fluency in older adults (Yu et al., 2025).

Although age-related differences in speech production have been well documented, their relationship with age-related variations in brain function remains largely unexplored. Compared to younger adults, older adults typically engage broader brain networks, often showing increased bilateral prefrontal activation (Cabeza, 2002; Davis et al., 2008). These age-related differences in activation patterns may reflect neural dedifferentiation (Li et al., 2001) or compensatory mechanisms (Cabeza et al., 2018). Age-related differences in brain function can be captured by resting state brain activation, reflecting how different brain regions activate and interact when a person is not engaged in any specific task (Friston, 1994). Correlated activation between regions during such task-free states forms intrinsic functional networks. Well-established networks include the default mode network, frontal-parietal network, and salience network (e.g., Chan et al., 2014; Power et al., 2011). Prior work has also identified a left-lateralized language network, comprising frontal and temporal regions that support core language processes (Fedorenko & Thompson-Schill, 2014; Ferstl et al., 2008).

In a healthy brain, the connectivity among nodes within a network is typically stronger than between networks. Network segregation—a measure that quantifies the difference between within- and between-network connectivity relative to within-network connectivity—captures the integrity and efficiency of intrinsic network organization (Chan et al., 2014). Older adults generally exhibit lower network segregation than younger adults, reflecting reduced network specialization and efficiency, which has been linked to declines in cognitive abilities (Chan et al., 2014, 2018; Zhang, Gertel, et al., 2021). Regarding language function, previous research has shown that higher segregation in the language network, as well as across the whole brain, is associated with better language production abilities in younger adults, but not in older adults (Zhang & Diaz, 2023).

Beyond functional connectivity, adults in different ages also exhibit differences in the magnitude of spontaneous low-frequency fluctuations in the blood oxygen level dependent (BOLD) signal (i.e., amplitude of low-frequency fluctuations [ALFF]; Zang et al., 2007; Zhang, Bai, & Diaz, 2021). Higher ALFF in key brain regions has been associated with superior behavioral performance in cognitive tasks (Mennes et al., 2011; Wei et al., 2012). Accordingly, older adults show reduced resting state ALFF in specific brain regions, and these reductions have been associated with cognitive decline in older adults (Fan et al., 2022; Jin et al., 2024; Ren et al., 2015). For instance, Ren et al. (2015) reported that higher ALFF in the parahippocampal gyrus was linked to better associative memory performance in older adults, while Fan et al. (2022) identified a direct positive relationship between ALFF and attention. Given that speech production depends not only on network-level coordination but also on the activation strength of specific brain regions involved in language (e.g., motor, auditory, and frontal cortices), examining resting state ALFF may provide additional insight into the neural mechanisms that support speech production in aging. Despite growing evidence linking resting state brain dynamics to cognitive performance, the relationship between resting state ALFF and speech production remains underexplored.

Individual differences in resting state brain activation and cognition can also be examined using representational similarity analysis (RSA), which is a multivariate technique that compares similarity across individuals based on patterns of brain activity or behavioral performance (Finn et al., 2020; Kriegeskorte et al., 2008). RSA is particularly well suited for studying high-dimensional data, such as whole-brain functional connectivity matrices, as it does not require strong assumptions about the form of brain–behavior relationships. Instead of modeling direct associations, this pattern-based approach assesses whether individuals with similar neural representations also exhibit similar cognitive profiles (Qiu et al., 2024). Specific to language-related processing, Devereux et al. (2013) used RSA in younger adults to reveal overlapping and distinct neural representations of object concepts presented as words and pictures. Specifically, they found that the left intraparietal sulcus showed modality-invariant representations, with word and picture representational dissimilarity matrices (RDMs) being significantly correlated. On the other hand, the word and picture RDMs in the left posterior middle temporal gyrus were not correlated, suggesting modality-specific semantic processing in this region. With regard to aging, a study found that brain representations of animacy in healthy aging exhibit increased similarity across categories, aligned with the neural dedifferentiation hypothesis of aging (Li et al., 2001; Margolles & Soto, 2024). Although RSA has proven to be a valuable approach for linking brain activity and cognitive processes, its application to understanding age-related differences in speech production remains largely unexplored.

In summary, resting state brain activity reflects intrinsic activation patterns in the absence of a task and is influenced by age. These age-related differences may contribute to cognitive differences across the life-span. While previous studies have primarily examined either functional connectivity or activation intensity, few have taken a more comprehensive approach by integrating both aspects to characterize brain states. Additionally, while much research has focused on memory, attention, and executive function, relatively little has examined age-related differences in speech production. Lastly, while younger adults’ brains tend to be highly specialized (e.g., language networks predominantly supporting language functions), aging often leads to reduced specialization, with more widespread brain networks contributing to behavior.

This study examines how aging affects resting state functional activity and speech production and how brain states—considering both connectivity and activation intensity—relate to behavioral performance. We hypothesize that older adults will exhibit more heterogeneous functional connectivity patterns, lower brain network segregation, and reduced activation intensity, alongside lower speech quality (e.g., reduced complexity and fluency). Furthermore, we predict that age-related differences in resting state brain activity will contribute to variability in speech performance.

## MATERIALS AND METHODS

### Participants

A total of 38 younger adults (27 female, 18–26 yr, Mean = 21.08 yr, *SD* = 1.94 yr), and 24 older adults (20 female, 61–76 yr, Mean = 67.92 yr, *SD* = 3.84 yr) were included in this study. All participants were community-dwelling individuals. Younger adults were recruited from local universities and nearby communities, and older adults were recruited from local community centers. Recruitment procedures were designed to ensure that both groups were drawn from similar community-based contexts. All participants were healthy, right-handed, and reported no history of neurological or physiological disorders, or any major medical conditions. Additionally, no participants reported taking any psychotropic medications that might affect the brain or cerebral blood flow. All participants had normal or corrected-to-normal vision, which was measured using the Freiburg Visual Acuity and Contrast Test (Bach, 1996), as well as normal color vision, which was measured using Ishihara Plates (Clark, 1924). All participants were native Cantonese speakers. Some participants reported speaking another language, but all reported living in an environment where the native language (L1; Cantonese) was dominant. All participants reported being highly dominant in their L1, with self-rated L1 proficiency higher than for any other language (*p* < 0.001). All participants provided written informed consent, and all experimental procedures were approved by the Research Ethics Committee of University of Macau.

### Data Acquisition

#### Neuropsychological testing

Preceding the magnetic resonance imaging (MRI) session, each participant underwent a series of psychometric and neuropsychological assessments designed to evaluate their basic cognitive profiles, including processing speed, executive function, memory, and language abilities. The screening tasks included the Montreal Cognitive Assessment (MoCA; Hong Kong version) to screen out mild cognitive impairment or dementia (Nasreddine et al., 2005); a geriatric depression scale with 15 items (GDS-15; a shortened version of GDS; score <7) to screen for individuals with depression (de Craen et al., 2003; Ferraro & Chelminski, 1996). Cognitive assessments included a forward and backward digit span task from the Wechsler Adult Intelligence Scale—Third Edition (WAIS-III) to assess working memory (Wechsler, 1997), and a simple reaction time test (i.e., respond to a white square as quickly as possible) to assess processing speed. Language assessment tasks included a Chinese version of the WAIS-III vocabulary subtest to measure vocabulary knowledge (Dai et al., 1990), a reading habits questionnaire (Acheson et al., 2008), and a categorical verbal fluency task (VF) to assess lexical retrieval (Filippetti & Allegri, 2011; Friesen et al., 2015; Malek et al., 2013). The neuropsychological testing results are shown in Table 1.

**Table 1.** Participants’ demographic and neuropsychological testing scores

	Younger adults	Older adults
	Mean (*SD*)	Mean (*SD*)
Age	21.08 (1.94)	67.92 (3.84)
Gender (f/m)	27/11	20/4
Education years	14.97 (1.85)	11.79 (4.45)**
Cognitive assessments		
MoCA (out of 30)	28.68 (1.23)	25.54 (2.57)***
Digit span forward	9.18 (2.86)	6.58 (1.41)***
Digit span backward	5.89 (1.78)	3.79 (1.77)***
Simple speed (ms)	273.98 (47.00)	320.43 (89.23)*
WAIS-III vocabulary	27.08 (5.72)	22.75 (6.21)**
Reading habits (out of 25)	20.92 (4.25)	19.71 (4.75)^ns^
VF (valid tokens)	13.24 (4.43)	12.71 (3.42)^ns^

*Note*. Age group difference was marked as: ***p < 0.001, **p < 0.01, *p < 0.05; ns indicates not significant.

#### Free speech task

In addition to the neuropsychological tasks, all participants performed a free speech task in Cantonese. This task was used to elicit unrestricted verbal responses. During the task, participants were asked to generate free speech on the topic, “What do you like or dislike about living in [a city they live in]?” Participants were given 15 s to think about the topic, then a 3 min period to articulate their thoughts, allowing for a natural and unstructured expression of ideas. Participants’ spoken responses were recorded using an audio recorder.

#### Acquisition of fMRI data

MRI data were collected on a 3T Siemens Prisma MRI scanner with a 32-channel head coil. We collected a sagittal T1 weighted localizer image to define a volume for data collection and higher order shimming. The anterior and posterior commissures were identified for slice selection and shimming. T1 weighted structural images were then collected using a magnetization-prepared rapid acquisition gradient echo (MPRAGE) sequence (repetition time [TR] = 2,300 ms; echo time [TE] = 2.28 ms; inversion time [TI] = 900 ms; flip angle = 8°; echo spacing = 7 ms; acceleration factor = 2; field of view [FOV] = 256 mm^2^; voxel size = 1 × 1 × 1 mm; 160 contiguous slices).

Resting state functional images sensitive to BOLD contrast were collected using an echo-planar imaging (EPI) sequence (TR = 2,500 ms; TE = 25 ms; flip angle = 90°; echo spacing = 0.49 ms; FOV = 240 mm^2^; voxel size = 3 × 3 × 3 mm; 41 contiguous axial slices, parallel to the anterior commissure–posterior commissure line, interleaved acquisition, 240 volumes). Two additional volumes were acquired and deleted at the beginning of each functional run to reach steady state equilibrium. Six runs of task-engaged functional images (128 volumes, 320 s for each) were also collected, yet they are not analyzed in the current study.

A field map sequence was also collected using a double-echo spoiled gradient echo sequence (TR = 446 ms; TE = 4.92 ms; flip angle = 63°; FOV = 216 mm^2^; voxel size = 2.4 × 2.4 × 2.4 mm; 60 contiguous slices; phase encoding = anterior to posterior, fat saturation = off; duration = 1: 12 min) that generated two magnitude images and one phase image.

### Data Coding

#### Speech data coding

**Figure 1.**
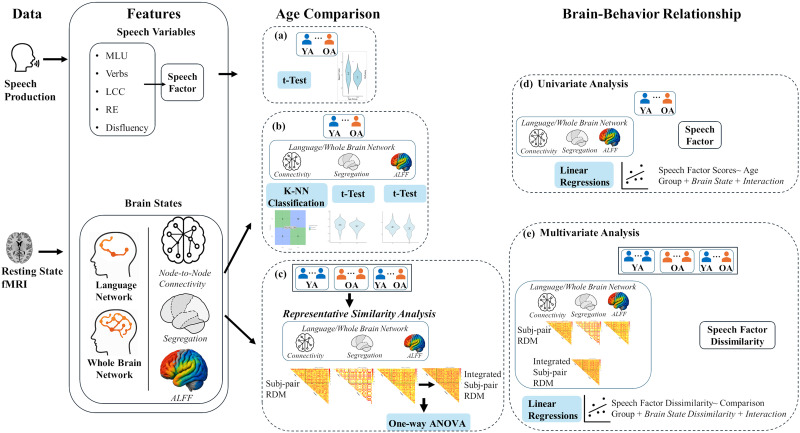
Overview of data processing and analyses. Speech production performance was indexed by a composite speech factor derived from five variables: mean length of utterance (MLU), number of verbs per utterance (Verbs), largest connected component (LCC), repeated edges (RE), and proportion of disfluencies (Disfluency). Resting state brain activity was characterized using node-to-node functional connectivity, network segregation, and amplitude of low-frequency fluctuations (ALFF) in both the language network and the whole-brain network. (A–C) Age group differences were examined through independent sample *t* tests on the speech factor, *t* tests on brain state measures, and representational similarity analysis of brain state measures. (D–E) The relationship between resting state activity and speech performance was further assessed through univariate regression on individual scores and multivariate regression on pair-wise individual dissimilarity scores. K-NN = k-nearest neighbors; RDM = representational dissimilarity matrix; YA = younger adults; OA = older adults.

All data processing and analysis steps are demonstrated in Figure 1. The coding and analyses approach for speech adhered to a previous study (Yu et al., 2025). Briefly here, speech recordings were first transcribed using the Computerized Language Analysis (CLAN; Version 25) software (MacWhinney, 2000) by one trained research assistant. According to CLAN guidelines, the following events were marked as speech disfluencies: irregular pauses (e.g., periods of silence >1 s within a phrase, generally shorter than 2 s), filled pauses (e.g., “um,” “uh”), phonetic fragments (e.g., “re-” for “relocate”), rephrasing (e.g., “and I drove—no, I walked to […]”), elongated words (e.g., “soooo”), simple repetition (e.g., “the the cat”), unintelligible speech, and errors that were not self-identified and corrected. Utterance boundaries were determined using a meaning-based segmentation approach in combination with the CLAN guideline (>2 s pause), dividing sentences if they conveyed independent propositions and combining them if clauses were tightly connected in meaning. After transcription, the morphological layer was generated using the MOR function and built-in Cantonese lexicons in CLAN. If a word was not in the default lexicon but deemed a real word, it was manually added to the lexicon.

Speech quality was evaluated based on complexity and fluency across five variables. Specifically, complexity was measured by: mean length of utterances (MLU) reflecting syntactic length, calculated as the total number of morphemes divided by the total number of utterances; number of verbs per utterances (Verbs) reflecting syntactic richness, as well as two graph-based measures: largest connected component (LCC), the total number of nodes, that is, words in the largest subgraph of lexical structure—where each node is connected to every other node through at least one path, and repeated edges (RE), which is a sum of all edges linking the same pair of nodes, that is, repeated word pairs. Fluency was quantified as the percentage of disfluencies, including both typical (i.e., common in fluent speakers, such as filled pauses) and stutter-like disfluencies (i.e., associated with speech disorders, such as sound repetitions). Intra-rater reliability, assessed on a subset of 10 participants (6 younger and 4 older adults; ∼15% of the sample), was excellent for all variables (all intraclass correlation coefficients >0.9, all *p*s < 0.001).

To examine whether the various speech measures reflected a common underlying construct of speech quality, an exploratory factor analysis (EFA) was conducted across all participants. This approach was both conceptually and empirically motivated, as fluency and complexity often co-occur—particularly in older adults with reduced speech production (Horton et al., 2010; Kemper et al., 2003, 2009). The composite factor thus provides a meaningful and efficient way to capture individual differences in overall communicative ability without isolating specific linguistic features. The analysis included all previously described variables, with no missing data or outliers (based on Mahalanobis distances, probability <0.001). Multicollinearity concerns among the cognitive variables were minimal as assessed by variance inflation factor (<3.5) and the data were normally distributed. Prior to conducting the factor analysis, a Bartlett’s test was conducted to determine the correlation adequacy among the cognitive variables, and a Kaiser-Meyer-Olkin test (KMO; Kaiser, 1974) was conducted to determine the sampling adequacy. Results suggested that there was a substantial correlation among the cognitive variables (Bartlett test *p* < 0.001) and that the sample was adequate (KMO = 0.76 > 0.60; Kaiser, 1974), which motivated the factor analysis. The psych package in the R environment was used for the factor analysis (Revelle, 2015). All speech variables were standardized using the scale() function in the R environment ((score-mean)/sd). One factor was extracted based on the kaiser criterion, which suggests retaining factors with eigen values >1, accounting for at least as much variance as one original variable. The final model used the varimax rotation and accounted for 52% of the variance in the data (TLI: 0.65; CFI: 0.82; RMSR: 0.11; RMSEA: 0.27). After identifying the latent speech factor, individual factor scores were calculated for each participant, with the overall mean equal to 0.

#### fMRI data preprocessing

Data quality was initially evaluated using the fBIRN QA tool (Glover et al., 2012), which assesses the number of voxels potentially affected by clipping, the mean signal fluctuation-to-noise ratio, and per-slice variability. Furthermore, anatomical and functional scans were manually inspected for artifacts and signal dropout. Preprocessing was conducted using the CONN functional connectivity toolbox (Version 18.a) within the MATLAB environment (Whitfield-Gabrieli & Nieto-Castanon, 2012). The pipeline included realignment and unwarping to correct for participant motion, distortion correction via a voxel displacement map derived from field maps, and slice-timing correction to account for the temporal evolution of the BOLD signal (Huettel et al., 2004). Outlier detection was conducted using the artifact detection tools method (NITRC, 2015), with outliers identified based on a conservative threshold (97th percentile) and subsequently excluded.

All structural and functional images were transformed into the standard Montreal Neurological Institute (MNI) space. Structural images were segmented into gray matter, white matter, and cerebrospinal fluid using the unified segmentation and normalization procedure in SPM12 (Ashburner & Friston, 2005), and these segmentations were applied to functional data. During coregistration, functional scans were aligned with structural images before both were normalized to standard space. A smoothing kernel of 6 mm was applied to enhance the signal-to-noise ratio and minimize voxel-level noise.

Denoising procedures included regressing out nuisance signals from white matter (5 components) and CSF (5 components) using the CompCor method, which extracts multiple components from non-neuronal regions to account for motion and physiological artifacts while preserving neural signals (Chai et al., 2012; Liu et al., 2017, 2021). A band-pass filter (0.008–0.09 Hz) was applied to remove unwanted frequency components (Davey et al., 2013; Gohel & Biswal, 2015; Hallquist et al., 2013). To control for potential confounds in data analysis, quality assurance metrics—including the number of outlier and non-outlier scans (outlier threshold = 0.5 mm), maximum and mean motion, and global BOLD signal fluctuations (outlier threshold = global signal *z* value of 3)—were accounted for. The average number of invalid scans was 1.4 out of 240 (0.6%, *SD* = 4.7) for younger adults and 5.1 out of 240 (2.1%, *SD* = 11.7) for older adults, with no significant age difference (*t*(27.86) = 1.45, *p* = 0.16). Mean head motion was 0.10 mm (*SD* = 0.03 mm) for younger adults and 0.16 mm (*SD* = 0.07 mm) for older adults, with a significant age effect (*t*(28.17) = 3.85, *p* < 0.001). All nuisance regressors were removed in a single linear regression step, and the residualized BOLD signal was used for further statistical analyses.

#### Node definition and network measures

We created a total of 264, 5 mm radius nonoverlapping nodes, using the MNI152 2 mm brain template, consistent with Power et al. (2011). Power et al. classified these nodes into 12 large-scale functional networks, including hand somatomotor, mouth somatomotor, visual, salience, auditory, cingulo-opercular control, frontoparietal control, ventral attention, dorsal attention, default mode, subcortical, and cerebellar networks. However, 33 nodes were excluded from the analysis due to poor classification alignment with Power’s original network assignments, leaving 231 nodes belonging to 12 networks across the whole brain (for MNI coordinates of all node locations, see Table S1 in the Supporting Information, available at https://doi.org/10.1162/NOL.a.208). Among the 231 nodes, nine nodes in the left hemisphere associated with language functions were further identified based on language parcellations established by Fedorenko et al. (2010), which were functionally defined (sentences > nonwords) and shown to be broadly involved in language processing. Another nine nodes located in the right hemisphere homologous regions were identified as belonging to the right language network. Although initially included to explore interhemispheric dynamics, the right language network was not analyzed independently in this study and reported here for completeness. Therefore, a total of 231 nodes belonging to 14 networks were identified with each node belonging to only one network and none overlapping (Zhang & Diaz, 2023). We specifically focused on the left language network connectivity (hereafter the language network) and the whole brain connectivity in the current study.

For each participant, the resting state time series were extracted for each node, and pair-wise cross-correlations were computed between the time courses of all node pairs, using CONN toolbox. Fisher’s transformation was applied to convert correlation coefficients into Z-values. Following prior studies that employed similar methodologies (Chan et al., 2014), negative correlations were excluded from further analysis due to ongoing debate regarding their interpretability (Hallquist & Hillary, 2018). The resulting connectivity matrix for each participant was a 231 × 231 weighted *z* matrix for the whole brain, and a 9 × 9 matrix for the left language network, with the diagonal and any negative values set to zero. To further reduce redundancy while retaining all unique pair-wise connectivity values, only the upper triangular portion of each node-to-node correlation matrix was extracted. These correlation matrices were subsequently imported into R software (Version R 4.3.1) for additional processing (Posit Team, 2025).

From a system-level approach, node-to-node connectivity was further converted to network segregation, using the approach used in previous studies (Chan et al., 2014; Zhang & Diaz, 2023). Specifically, for all the 14 networks described above, within-network connectivity was computed as the mean correlation among all nodes within a given network, while between-network connectivity was determined as the mean correlation between nodes in one network and those in all other networks. The network segregation value was calculated using the formula (within-between)/within. For each participant, the language network segregation was first calculated, and the whole brain network segregation was further obtained by averaging segregation values across all networks.

In addition to functional connectivity, the intensity of spontaneous brain activity during resting state was captured through the ALFF using the CONN toolbox. Briefly, for a given voxel, the filtered time course was first converted to the frequency domain using fast Fourier transform. The square root of the power spectrum was computed and then averaged across the frequency band (0.008–0.09 Hz). The averaged square root was referred to as the ALFF (Zang et al., 2007). For each participant, the ALFF of each node was calculated by averaging across all the voxels in this node, and the mean ALFF for each individual was calculated by averaging across all nine nodes in the language network, and all 231 nodes in the whole brain. The ALFF values were normalized before being entered into further analysis.

### Data Analyses

#### Main effects of age group

To examine age-related differences in speech quality, we first conducted an independent sample *t* test on the speech factor score derived from factor analysis. We then extended the analysis to resting state brain activity to further explore age effects.

To assess group differences in overall functional connectivity patterns, we employed a supervised, instance-based, nonparametric k-nearest neighbors (k-NN) classification approach. Specifically, functional connectivity patterns were converted to feature vectors within the language network and across the whole brain respectively for each participant. The extracted features were compiled into a single dataset, with each row corresponding to an individual participant and labeled according to their group membership. Then participants were classified into different groups using Euclidean distance based on the extracted features. Prior to classification, the dataset was randomly partitioned into training (70%, 45 participants) and testing (30%, 20 participants) subsets using stratified sampling to ensure balanced group representation. A k = 6 was used, as determined by the square root of the number of data points in the training set. The trained k-NN model was applied to the test dataset, and predicted group labels were compared against actual labels. Classification performance was assessed using a confusion matrix, which indicated the number of correctly and incorrectly classified participants in each group. The above-mentioned analysis was conducted on the left hemisphere language network, then across the whole brain separately.

To explore age group difference in network segregation, an independent sample *t* test was conducted on language network, and the whole brain respectively, while including Age Group as the predictor. One participant had a negative language network segregation value, indicating weaker within-language than between-network connectivity. This case was further identified as an outlier being outside of the 2.5 *SD* range of the group level language network segregation mean, therefore, was removed from the segregation-related analysis.

Similar independent sample *t* tests were conducted to explore the age effects on ALFF within the left hemisphere language network, and across the whole brain. One participant exhibited a whole-brain ALFF value that was negative and fell beyond 2.5 *SD* from the group mean. Given that ALFF is a measure of the amplitude of low-frequency fluctuations and is typically positive, to ensure the reliability of our results and maintain data integrity, we excluded this participant from ALFF-related analyses.

#### Individual differences in resting state brain activity

In addition to traditional analyses at the group level, individual dissimilarities were captured through a RSA (Kriegeskorte et al., 2008), a multivariate approach that complements univariate *t* tests by detecting subtle resting state brain state distinctions. First, based on the node level functional connectivity patterns within the language network and across the whole brain respectively for each participant, a representative pair-wise node dissimilarity matrix (RDM) was created across all participants. Dissimilarity between participants was defined as 1 − *r*, where *r* is the Pearson correlation between the vectorized upper triangular elements of their respective connectivity matrices. Then to explore node connectivity dissimilarities within and between age groups, a one-way analysis of variance (ANOVA) was conducted on normalized dissimilarities including comparison group as the predictor (within younger, within older, and between younger and older adults).

Furthermore, pair-wise individual dissimilarity values in network segregation were computed within the language network and across the whole-brain networks using Euclidean distance. A similar one-way ANOVA was conducted on normalized segregation dissimilarity to explore the effect of comparison group. Similarly, the pair-wise dissimilarity in ALFF was calculated for nodes within the language network, as well as for all nodes across the brain, also using Euclidean distance. Subsequently, a one-way ANOVA was conducted to explore the effect of comparison group.

Last but not the least, for the left language network and the whole brain, we integrated the three dimensions of dissimilarity by adding the scaled dissimilarity variable in each dimension then divided by 3 (i.e., [normalized node connectivity dissimilarity + normalized segregation dissimilarity + normalized ALFF dissimilarity]/3). Then a one-way ANOVA was conducted to explore the effect of comparison group.

#### Relationship between resting state functional activity and speech

To explore how resting state connectivity contributes to age-related differences in speech performance, several sets of analyses were conducted separately for the language network and the whole brain.

First, linear regressions were conducted with the individual speech factor scores (derived from the EFA of speech complexity and fluency measures) as the dependent variable. The model included Age Group, network segregation, and their interaction as predictors. Similarly, separate simple linear regressions were conducted using the individual speech factor score as the dependent variable, with Age Group, ALFF value, and their interaction as predictors. The age group was numerically coded (Younger as −0.5, Older as 0.5) and all continuous variables were standardized.

In addition to the traditional analysis, we explored how dissimilarity in resting state contributes to dissimilarity in speech. To analyze this relationship, we first captured dissimilarity in speech performance through the RSA analysis using Euclidean distances, creating a pair-wise dissimilarity matrix across all participants, keeping only the upper triangular unique comparisons. Greater distance indicated larger differences in speech factor scores between every pair of participants. Then we conducted a series of linear regressions on speech dissimilarity. Specifically, for each metric (i.e., node connectivity, network segregation, ALFF), we regressed pair-wise speech dissimilarity on dimensional (brain) dissimilarity, comparison group (within younger, within older, and between groups, numerically coded as −0.5, 0, 0.5, respectively), and their interaction. An additional regression was conducted on the speech dissimilarity that included integrated brain state dissimilarity, comparison group, and their interaction as predictors. All continuous variables were standardized.

To assess the robustness of our regression results given the modest sample size, we conducted permutation tests. This nonparametric approach evaluates whether observed brain–behavior associations could have occurred by chance. For each test, the outcome variable was randomly shuffled across participants, and the model was re-estimated over 1,000 iterations to generate a null distribution. Observed effects were compared against this distribution to obtain permutation-based *p* values. All data and analysis scripts can be found at https://osf.io/7f695/.

## RESULTS

### Main Effects of Age Group

#### Age effects on speech performance

**Figure 2.**
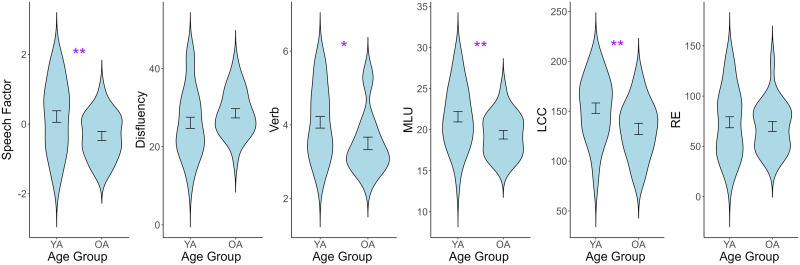
Age effects on speech performance. Older adults showed significantly lower speech factor scores than younger adults, driven by differences in complexity measures such as mean length of utterances (MLU), Verbs, and largest connected component (LCC). RE = repeated edges; YA = younger adults; OA = older adults.

The EFA identified a single speech factor that captured shared variance across all individual language variables. Specifically, the identified speech factor loaded positively on Verbs (loading = 0.63), MLU (loading = 0.81), LCC (loading = 0.76), RE (loading = 0.73), and negatively on Disfluency (loading = −0.67). Therefore, a higher speech factor score reflected better speech quality in general. An independent sample *t* test showed a significant main effect of Age Group (Figure 2), such that the factor score of older adults was lower than younger adults, *t*(59.99) = 2.67, *p* = 0.009. A follow up exploration showed that there were significant negative effects of age on language complexity measures including MLU, *t*(59.90) = 2.70, *p* = 0.009; Verbs, *t*(54.37) = 2.47, *p* = 0.02; LCC, *t*(54.25) = 2.71, *p* = 0.009; but there were no significant age differences for RE, *t*(58.90) = 0.58, *p* = 0.56, or Disfluencies, *t*(59.78) = 1.31, *p* = 0.20.

#### Age effects on functional connectivity and activation intensity

**Figure 3.**
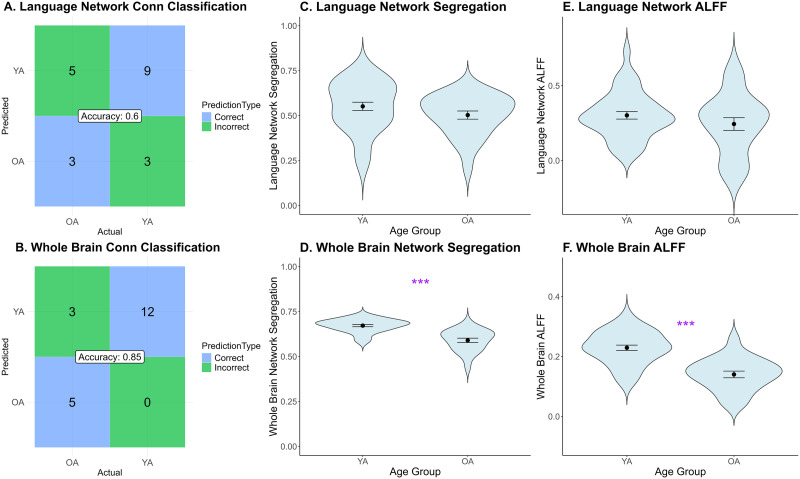
Age effects on resting state functional activity. The classification was first trained on 70% of participants then tested on the remaining 30% (*N* = 20) participants. The classification accuracy was 0.6 in (A) language network (only slightly above chance) and 0.85 in the (B) whole brain, indicating comparable language network connectivity between younger and older adults, but different whole brain connectivity between age groups. Main effect of age group was not significant on (C) language network segregation or (E) language network amplitude of low-frequency fluctuations (ALFF). Main effect of age group was significant on (D) whole brain network segregation and (F) whole brain ALFF. YA = younger adults; OA = older adults.

To explore the main effects of age on resting state brain activation in the language network and the whole brain, several sets of analyses were conducted. First, a classification test was conducted to evaluate whether node-level connectivity patterns are distinct enough between age groups, by training a k-NN model on part of the dataset and then testing how accurately it can predict group membership on new data (k = 6, decided by square root of the number of data points *N* = 45 in the training set). After classification training, the classification test was conducted on the testing group (*N* = 20, including 8 older and 12 younger adults). For within language network connectivity (9 × 9 correlation matrix), three older and nine younger adults were correctly classified. There were five misclassifications for older adults and three for younger adults. The overall classification accuracy was 60%, only slightly above chance level, demonstrating limited success in predicting age group membership based on within language network connectivity, indicating the stability of language network with age (Figure 3A). For whole brain network connectivity (231 × 231 correlation matrix), 5 older and 12 younger adults were correctly classified. There were three misclassifications for older adults. The overall classification accuracy was 85%, demonstrating a high level of accuracy in predicting age group membership based on whole brain node connectivity, indicating strong age difference on the whole brain level (Figure 3B).

A second way we looked at age differences is through a system perspective, where independent sample *t* tests were conducted on network segregation, reflecting how functionally specialized networks are. The effect of age was not significant on language network segregation, such that the language network structure was comparable between two age groups, *t*(56.39) = 1.51, *p* = 0.14 (Figure 3C). The effect of age on whole brain network segregation was significant, indicating that older adults’ whole brain was less segregated than younger adults, *t*(34.60) = 6.07, *p* < 0.001 (Figure 3D).

Third, we examined age differences in resting state brain activity by analyzing ALFF, which indexes the intensity of spontaneous neural activity, both within the language network and across the whole brain. Independent sample *t* tests showed no significant effect of age on language network ALFF, *t*(37.34) = 1.18, *p* = 0.25 (Figure 3E), but a significant effect of age group on whole brain ALFF, which was higher in younger than older adults, *t*(47.36) = 6.24, *p* < 0.001 (Figure 3F).

#### Dissimilarity in functional connectivity and activation intensity

**Figure 4.**
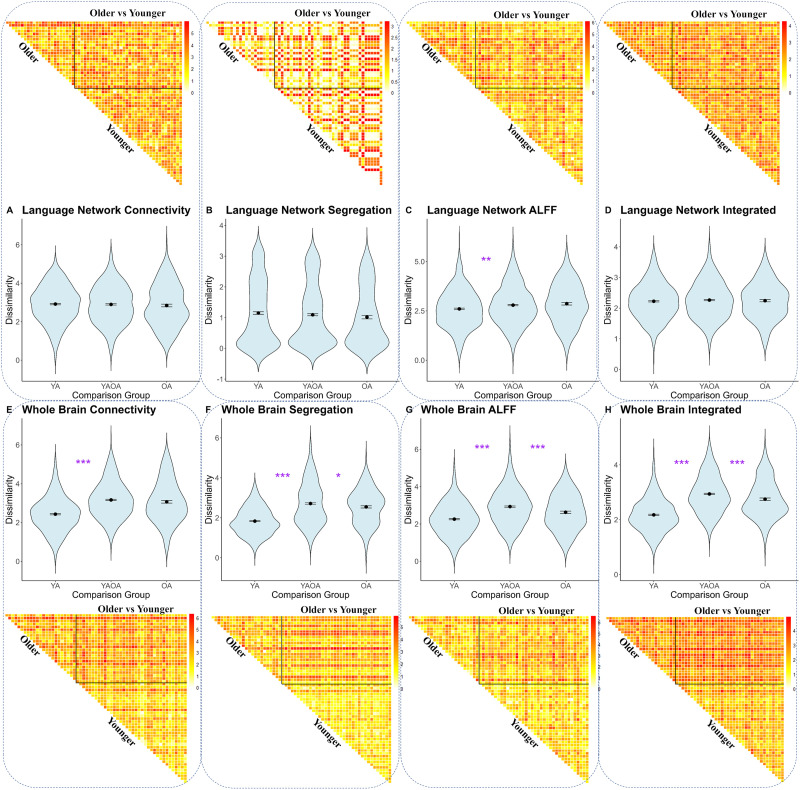
Dissimilarity in functional connectivity and activation intensity. The upper panels (A–D and the top heat maps) indicate dissimilarity patterns within the left language network, with comparable dissimilarity patterns within younger group, within older group, and between age groups. The lower panels (E–H and the bottom heat maps) indicate dissimilarity patterns across the whole brain. Color bars in the heat maps indicate pair-wise dissimilarity with darker red representing higher dissimilarity and lighter yellow presenting lower dissimilarity. Overall, younger adults were more homogenous to each other than older adults with each other (i.e., lower right triangles lighter than upper left triangles). Additionally, participants within the same age group were more homogenous than participants between age groups (i.e., triangles lighter than the upper right square). YA = younger adults; OA = older adults; YAOA = younger vs. older adults; ALFF = amplitude of low-frequency fluctuations.

In addition to the direct age group comparison on different indicators of resting state brain profile, we further explored dissimilarity in these measures by using RSA methods. First, pair-wise dissimilarity on node level connectivity was calculated within the language network and across the whole brain among all participants. Then we explored the within and between age-group differences using a one-way ANOVA. With the language network, there was no significant effect of comparison group (*F*(2, 1,767) = 0.50, *p* = 0.61; Figure 4A). However, for the whole brain connectivity patterns, there was a significant effect of comparison group (*F*(2, 1,767) = 120.2, *p* < 0.001; Figure 4E), such that the dissimilarity patterns within younger adults were lower than that within the older adult group (*p* < 0.001). Additionally, participants within the younger adult group had less dissimilar connectivity patterns than between age groups (*p* < 0.001). These results suggest that while there was no group difference in the language network, younger adults exhibit more homogeneous connectivity patterns across the whole brain, while older adults and the comparisons between age groups display more heterogeneous connectivity patterns.

With network segregation, we calculated pair-wise dissimilarity across participants within the language network and across all networks in the whole brain using Euclidean distance. Then we explored the within and between age-group differences using one-way ANOVAs. With the language network segregation, there was no significant effect of comparison group (*F*(2, 1,767) = 1.71, *p* = 0.18; Figure 4B). Regarding the whole brain network segregation patterns, there was a significant effect of comparison group (*F*(2, 1,767) = 180.3, *p* < 0.001; Figure 4F), such that participants within the younger adult group had less variable connectivity patterns than within older adults (*p* < 0.001). Additionally, the between-group connectivity dissimilarity was higher between groups than within the younger adult group (*p* < 0.001) and within the older adult group (*p* = 0.03). These results indicate that whole brain network segregation was more homogeneous and higher within younger adults than older adults.

With ALFF, similar pair-wise dissimilarity matrices across participants were calculated from the Euclidean distance of all nodes in language network, and across the whole brain. Within the language network, there was a significant effect of comparison group (*F*(2, 1,767) = 9.53, *p* < 0.001; Figure 4C), such that participants within the younger group showed significantly lower dissimilarity than participants within older adults (*p* = 0.001), and were less variable than between group comparison (*p* < 0.001). Across the whole brain, there was a significant effect of comparison group (*F*(2, 1,767) = 93.42, *p* < 0.001; Figure 4G), such that younger adults were significantly less variable than older adults (*p* < 0.001), which was significantly lower than the dissimilarity between groups (*p* < 0.001).

Finally, we combined three levels of dissimilarity (node-wise functional connectivity, network segregation, and ALFF) into an integrated participant pair-wise dissimilarity measurement for the language network and the whole brain network. Within the language network, there was no significant effect of comparison group (*F*(2, 1,767) = 0.77, *p* = 0.46; Figure 4D). Across the whole brain, there was a significant effect of comparison group (*F*(2, 1,767) = 257.3, *p* < 0.001; Figure 4H). Further analysis showed that the integrated dissimilarity within the younger adult group was significantly lower than within the older adult group (*p* < 0.001). Additionally, the within age group dissimilarity in either group was also significantly lower than the dissimilarity between age groups, which was lower than between group comparison (*p*s < 0.001).

### Relationship Between Resting State Brain Activity and Speech

#### Relationship between network segregation and speech

**Figure 5.**
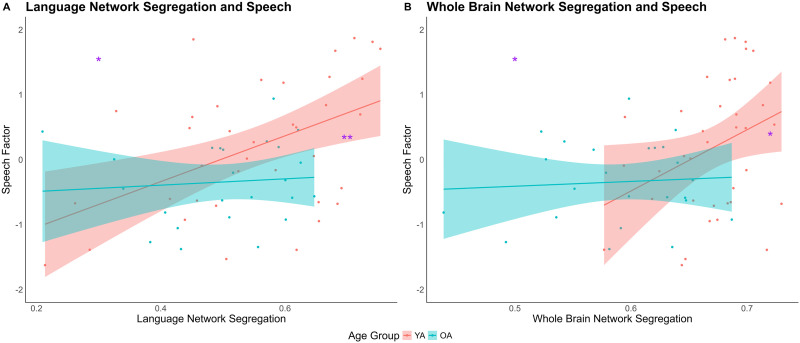
Relationship between network segregation and speech. The (A) language network segregation and the (B) whole brain network segregation positively relate to speech production, driven by younger adults. YA = younger adults; OA = older adults.

In addition to age effects on network segregation, we further explored the relationship between network segregation and speech in different age groups through simple linear regressions. Specifically, the model included the speech factor as the dependent variable, while including Age Group, Network Segregation, and their interactions as predictors. In the model including only the language network (Figure 5A), there was a main effect of Age Group, *β* = −0.46, *t* = −2.08, *p* = 0.04, and a main effect of language network segregation, *β* = 0.26, *t* = 2.19, *p* = 0.03. Permutation tests confirmed the significance of both effects (Age Group, *p* = 0.03; language network segregation, *p* = 0.03). The interaction between Age Group and language network segregation was not significant (*β* = −0.39, *t* = −1.66, *p* = 0.10). Although not quite significant, we explored the involvement of language network segregation in each age group separately. Results showed a positive relationship between language network segregation and speech factor in younger (*β* = 0.49, *t* = 3.22, *p* = 0.003) but not in older adults (*β* = 0.05, *t* = 0.40, *p* = 0.69). Further comparison showed that the regression coefficients difference between younger and older adults was significantly different (*z* = 2.15, *p* = 0.03).

In the model including overall whole brain network organization (Figure 5B), there was a main effect of whole brain network segregation, *β* = 0.31, *t* = 2.04, *p* = 0.046, further confirmed by a nonparametric permutation test (*p* = 0.049), indicating that higher whole brain network segregation was significantly correlated with better speech. Additionally, the interaction between Age Group and whole brain network segregation was marginally significant, *β* = −0.53, *t* = −1.74, *p* = 0.09, and this effect was further supported by a nonparametric permutation test (*p* = 0.07). Further follow-up analyses showed that the positive relationship between network segregation and speech was only significant in younger adults, *β* = 0.35, *t* = 2.12, *p* = 0.04, but not in older adults, *β* = 0.04, *t* = 0.33, *p* = 0.75. Further comparison showed that the regression coefficients difference between younger and older adults were not significantly different (*z* = 1.43, *p* = 0.15).

#### Relationship between activation intensity and speech

**Figure 6.**
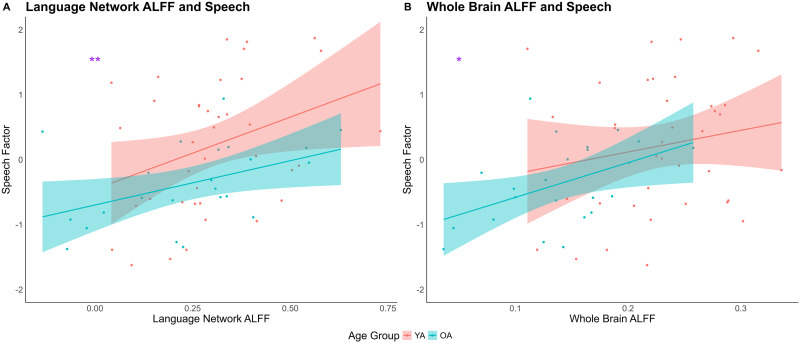
Relationship between amplitude of low-frequency fluctuations (ALFF) and speech. Across both age groups, higher ALFF in (A) language network and (B) whole brain were associated with better speech production. YA = younger adults; OA = older adults.

In addition to the age effects on ALFF, we further explored the relationship between the language and whole brain network ALFF, speech, and Age Group through two separate linear regressions. In the first regression (Figure 6A), we found a significant main effect of Age Group (*β* = −0.48, *t* = −2.12, *p* = 0.04), a main effect of language network ALFF on the speech factor, such that higher ALFF in language network was associated with higher speech factor scores (*β* = 0.31, *t* = 2.82, *p* = 0.007). Permutation tests confirmed the significance of both effects (Age Group, *p* = 0.03; language network ALFF, *p* = 0.007). In the second regression (Figure 6B), the effect of whole brain ALFF on speech factor scores was nearly significant (*β* = 0.30, *t* = 1.98, *p* = 0.053), consistent with a nearly significant nonparametric permutation test (*p* = 0.055), such that higher whole brain ALFF was associated with higher speech factor scores. In both regressions, the interactions between ALFF and Age Group were not significant (*p*s > 0.1).

#### Relationship between dissimilarity in resting state brain activity and speech

**Figure 7.**
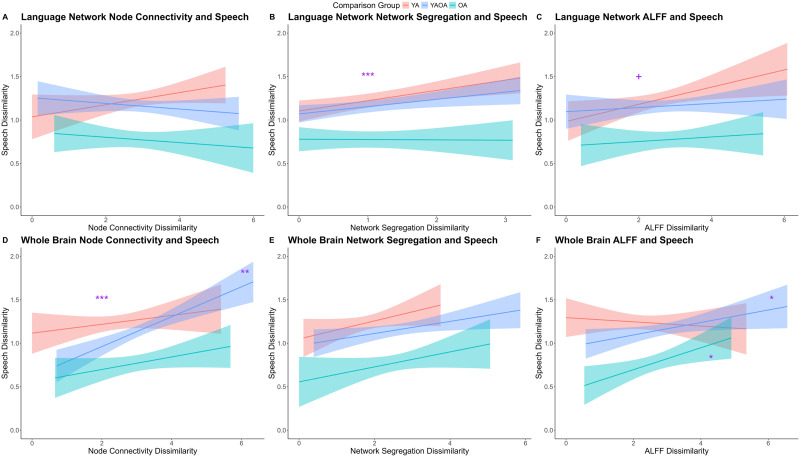
Relationship between dissimilarity in resting state brain activity and speech. Regarding the language network, top panels show: (A) Significant interaction between dissimilarity in node connectivity and dissimilarity in speech. (B) Significant relationship between dissimilarity in segregation and dissimilarity in speech. (C) No significant relationship between dissimilarity in ALFF and dissimilarity in speech, although no significant relationship was found in either group. Regarding the whole brain, bottom panels show: (D) Significant relationship between dissimilarity in node connectivity and dissimilarity in speech, driven by the comparison between younger and older adults. (E) Significant relationship between dissimilarity in network segregation and dissimilarity in speech. (F) Significant relationship between dissimilarity in amplitude of low-frequency fluctuations (ALFF) and dissimilarity in speech when comparing between younger and older adults and comparing within older adults. YA = younger adults; OA = older adults; YAOA = younger vs. older adults.

We first explored whether the dissimilarity in node-to-node resting state functional connectivity would contribute to dissimilarity in speech performance in different groups. A simple linear regression was first conducted on speech factor scores, including the node-to-node inter-participant dissimilarity within the language network connectivity, comparison group (within younger, within older, and younger vs. older), and their interactions as predictors (Figure 7A). Although the main effects were not significant (*p*s > 0.1), the interaction between comparison group and node connectivity dissimilarity within the language network RDM was marginally significant (*β* = −0.10, *t* = −1.93, *p* = 0.05), further confirmed by a nonparametric permutation test (*p* = 0.056). Figure 7A shows that the node connectivity dissimilarity was trending toward a positive relationship with speech dissimilarity in younger adults, but toward a negative relationship with speech dissimilarity in older adults and between-group pairs, although none of these effects reached significance (*p*s > 0.1). A similar regression was conducted on speech score, including the interparticipant node connectivity dissimilarity across the whole brain, comparison group, and their interactions as predictors (Figure 7D). Results showed a significant effect of comparison group (*β* = −0.63, *t* = 3.90, *p* < 0.001; supported by a nonparametric permutation test, *p* < 0.001), a main effect of whole brain connectivity dissimilarity (*β* = 0.08, *t* = 3.33, *p* < 0.001; supported by a nonparametric permutation test, *p* < 0.001), such that more dissimilar whole brain connectivity was associated with more dissimilar speech. Additionally, there was a significant interaction between the comparison group and whole brain connectivity dissimilarity (*β* = 0.18, *t* = 3.27, *p* = 0.001; supported by a nonparametric permutation test, *p* = 0.002). Further analyses showed that only when comparing younger and older adults, but not when comparing within each age group, the relationship between whole brain connectivity dissimilarity and speech dissimilarity was significant (*p* < 0.001). Analyses comparing the beta values further indicated that the brain–behavior relationship when comparing between younger and older adults was significantly stronger than within younger adults (*z* = 2.06, *p* = 0.04) and marginally significantly stronger than within older adults (*z* = 1.75, *p* = 0.08).

Second, we explored whether the dissimilarity in network segregation across participants would relate to the dissimilarity in speech performance. A simple linear regression was first conducted on speech dissimilarity including the within language network segregation, comparison group, and their interaction (Figure 7B). The main effect of language network segregation dissimilarity was significant (*β* = 0.09, *t* = 3.94, *p* < 0.001), further confirmed by a nonparametric permutation test (*p* < 0.001). The main effect of comparison group, and the interaction between the two were not significant (*p*s > 0.1). These results suggest that the dissimilarity in language network segregation was positively associated with speech dissimilarity across all participants. Another regression including the whole brain network segregation dissimilarity showed no significant effect of comparison group, whole brain segregation, or their interaction (*p*s > 0.06; Figure 7E).

Third, we explored whether the dissimilarity in ALFF would contribute to the dissimilarity in speech across participants. The simple linear regression including the language network showed a marginally significant effect of ALFF dissimilarity on speech dissimilarity (*β* = 0.04, *t* = 1.89, *p* = 0.06; Figure 7C), further supported by a nonparametric permutation test (*p* = 0.06). There was no significant main effect of comparison group or interaction between the two (*p*s > 0.1). The linear regression including the whole brain ALFF showed a significant main effect of comparison group (*β* = −0.44, *t* = −2.92, *p* = 0.004; supported by a nonparametric permutation test, *p* = 0.004), and its interaction with comparison group (*β* = 0.13, *t* = 2.45, *p* = 0.01; supported by a nonparametric permutation test, *p* = 0.013; Figure 7F). Follow-up analysis showed that only when comparing between younger and older adults (*β* = 0.07, *t* = 2.13, *p* = 0.03) and within older adults (*β* = 0.13, *t* = 2.57, *p* = 0.01) was the relationship between whole brain ALFF dissimilarity and speech dissimilarity significant. Yet, this relationship within younger adults was not significant (*p* = 0.60). Further analyses indicated that the ALFF-speech relationship within older adults was indeed significantly stronger than that within younger adults (*z* = 2.21, *p* = 0.03) but not significantly different between age groups and within younger adults (*z* = 1.66, *p* = 0.096). This suggests that whole brain ALFF was closely related to speech performance in older adults but not in younger adults.

#### Relationship between integrated dissimilarity in resting state brain activity and speech

**Figure 8.**
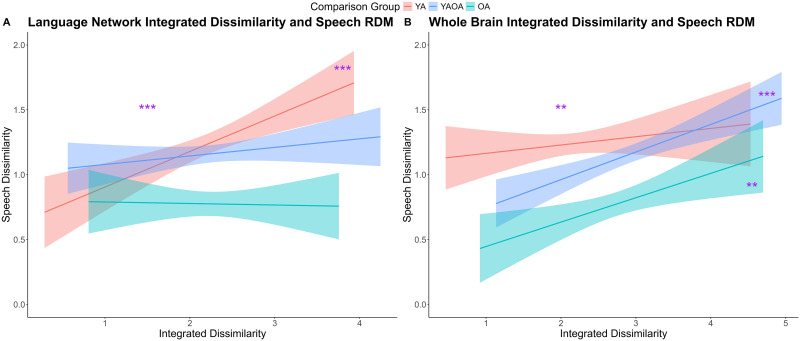
Relationship between integrated dissimilarity in resting state brain activity and speech. (A) Significant positive relationship between language network integrated dissimilarity and speech dissimilarity, driven by younger adults. (B) Significant positive relationship between whole brain integrated dissimilarity and speech dissimilarity, driven by comparison between younger and older adults, and comparison within older adults. RDM = representational dissimilarity matrix; YA = younger adults; OA = older adults; YAOA = younger vs. older adults.

As mentioned previously, we combined the RDM across different dimensions into one integrated RDM measurement, indicating the overall dissimilarity in the resting state brain activity across all participants. We further explored how this integrated resting state dissimilarity contributes to speech dissimilarity. The first regression focuses on the language network, including the speech dissimilarity as the dependent variable, while including the language network integrated dissimilarity, the comparison group, and their interaction as independent variables (Figure 8A). The main effect of integrated language network dissimilarity was positively significant, *β* = 0.14, *t* = 3.59, *p* < 0.001, and its interaction with comparison group was significant, *β* = −0.19, *t* = −2.24, *p* = 0.03. Permutation tests confirmed that both effects remained significant beyond parametric assumptions (integrated language network dissimilarity, *p* < 0.001; interaction with comparison group, *p* = 0.03). Follow-up analyses showed a positive relationship between language network dissimilarity and the speech factor only in younger adults (*β* = 0.27, *t* = 3.95, *p* < 0.001) but not when comparing across age groups or within older adults (*p*s > 0.1). Further analyses showed that the network–speech relationship in younger adults was indeed significantly stronger than that in older adults (*z* = 2.69, *p* = 0.007; see red line vs. green line in Figure 8A) and across age groups (*z* = 2.34, *p* = 0.02; see red line vs. blue line in Figure 8A).

In the model including overall whole brain resting state dissimilarity, the main effect of brain dissimilarity was positive and significant, *β* = 0.11, *t* = 3.06, *p* = 0.002, indicating that more dissimilar brain resting state was associated with more dissimilar speech performance (Figure 8B). The main effect of comparison group was significant (*β* = −0.82, *t* = −4.06, *p* < 0.001), along with its interaction with brain resting state dissimilarity (*β* = 0.26, *t* = 3.38, *p* < 0.001). Permutation tests further confirmed that all these effects remained significant beyond parametric assumptions (*p*s < 0.001). Further analyses showed that the relationship between brain resting state and speech was significant when comparing between younger and older adults (*β* = 0.21, *t* = 4.36, *p* < 0.001) and when comparing within older adults (*β* = 0.19, *t* = 2.74, *p* = 0.007), but not when comparing within younger adults (*β* = 0.06, *t* = 0.94, *p* = 0.35). An additional comparison on the beta values indicated that the brain–behavior relationship within younger adults was indeed weaker than that between younger and older adults (*z* = 1.77, *p* = 0.08; see red line vs. blue line in Figure 8B) but not significantly different in other comparisons on regression beta values (*p*s > 0.1).

## DISCUSSION

The current study investigated age-related differences in speech production and the extent to which resting state brain activity contributes to these differences. Speech performance was captured through a novel composite factor score using an integrated measurement of speech complexity and fluency. Older adults showed significantly lower speech factor scores compared to younger adults, reflecting overall reduced speech quality with age. The resting state functional connectivity and activation patterns were captured through multidimensional measures. Briefly, the language network shows stability with age, while the whole brain analysis shows significant age differences. Relating the results to production, resting state functional activity significantly relates to speech performance, but these brain–behavior relationships vary by the specific network and by age group. Univariate analysis revealed strong associations between brain state predictors and speech performance, primarily within younger adults. Multivariate RSA-based regressions highlighted a dissociative pattern, showing that brain–speech relationships were strongest within the language network for younger adults, but more pronounced in the whole-brain network when capturing individual differences in older adults. Below, we discuss these findings in detail.

For understanding speech performance at the behavioral level, we used a factor analysis approach to integrate multiple dimensions such as complexity and fluency of speech and found that older adults’ speech performance was overall worse than that of younger adults. Previous research also demonstrating age-related differences in speech has predominantly focused on single dimensions of speech without reflecting overall speech ability or quality. Acknowledging the value of an integrated speech measurement, we further explored age differences in each dimension and found that the age differences were mainly driven by the complexity measures such as length of utterances and number of verbs per utterance, indicating that older adults produced shorter and less complex sentences than younger adults. Consistent with previous findings (Altmann & Kemper, 2006; Kemper et al., 2003), this age difference may be related to the working memory demands associated with producing longer and more complex sentences. As older adults, both in this sample and generally, experience declines in working memory capacity (Kemper & Kemtes, 2002), they may find it more challenging to manage the cognitive load required for constructing and maintaining complex sentence structures.

Yet, there was no significant age difference in speech disfluency. In fact, although the age-related increase in disfluency was expected and has been previously reported (Bóna, 2014; Schmitter-Edgecombe et al., 2000), some studies have failed to find this pattern (Bóna, 2011; Cooper, 1990; Keszler & Bóna, 2019). One possible explanation for this finding is that older adults might be more conscious of their speech patterns and engage in greater self-monitoring, adopting compensatory strategies such as pausing longer between sentences or simplifying content to avoid disfluencies (Bóna, 2011). The absence of age-related increases in disfluency alongside simpler speech production in older adults may be related. Specifically, syntactic planning that involves producing shorter or simpler utterances—more typical in older adults—likely depends on well-practiced, automatic speech processes that are less prone to disfluency (Ivanova & Ferreira, 2019). Recent research has further emphasized the importance of distinguishing among disfluency types, as they may reflect different cognitive mechanisms (Beier et al., 2023; Clark & Tree, 2002; Engelhardt & Markostamou, 2025). For instance, repetitions are often linked to inhibition, while filled pauses are associated with working memory. Although subgroup analyses were beyond the current study’s scope, exploratory checks revealed no significant age differences across disfluency subtypes (*p*s > 0.1). The narrative recall task may have also promoted more structured responses, reducing opportunities for spontaneous disfluencies. Additionally, while all participants identified Cantonese as their native language, younger adults may be less proficient due to the rise of Mandarin in recent decades (Li et al., 2022). In contrast, older adults—more proficient in Cantonese and likely socialized to value fluency and politeness in formal speech—may exhibit lower disfluency rates (Lee, 2020), further attenuating age effects. Nevertheless, focusing on age-related difference in overall speech production, older adults exhibited lower speech quality compared with younger adults, reflected in sentence complexity.

In addition to speech production performance, we explored the age-related differences in resting state functional activation. Using novel methodologies, we captured resting state activation through multiple dimensions including functional connectivity and intensity, with complementary approaches including node-to-node weighted connectivity and network analysis. We found that the whole brain network segregation showed significant effects of age, while the language network segregation remained stable across age groups. This pattern is consistent with previous findings (Chan et al., 2014; Zhang & Diaz, 2023). In a previous study using an adult life-span sample (22–89 yr), researchers found that older age was associated with stable language network segregation, but lower whole brain network segregation (Zhang & Diaz, 2023). Extending this work, we demonstrated the dissociative patterns between the language network and the whole brain from not only network segregation measures, but also a classification test based on node-to-node connectivity patterns. In the language network, the classification accuracy was lower, indicating that it is hard to predict the age group based on functional connectivity patterns within the language network alone, suggesting less pronounced age differences or potentially a lack of differences altogether. On the other hand, in the whole brain node-to-node connectivity, the classification accuracy was higher, indicating that the two age groups were distinguishable based on whole brain connectivity, consistent with the network segregation patterns. Consistent with the findings on functional connectivity, older adults show lower amplitude low-frequency resting state fluctuations than younger adults in the whole brain, but not in the language network. These results are consistent with previous research reporting a global reduction in ALFF with aging, particularly in the right hemisphere and posterior cerebellum (Fan et al., 2022; Zhang, Xue, et al., 2021), which might be related to neuronal loss and brain atrophy in older adults. Overall, we found that across both connectivity and fluctuation amplitude, the whole brain functional activity showed significant effects of age, while the language network remained stable with age.

Additionally, through an RSA, we found two distinct patterns of age-related brain variability. First, the pair-wise dissimilarity was lower within the same age group (younger–younger, older–older) than between age groups (younger–older) across network segregation, ALFF, and the integrated measurement on the whole brain. These results indicate more homogenous brain states within each group, but more heterogenous states between age groups, further supplementing the above-reported age difference in the whole brain status. In contrast, this pattern was not found for the language network, indicating a comparable language network profile across age groups. Second, younger adults showed more homogenous whole-brain functional connectivity, whereas older adults showed more heterogenous connectivity (i.e., greater variability). This was reflected by lower within-group dissimilarity among younger adults compared to older adults across all metrics (see Figure 4E–H). One interpretation of these results is that younger adults are typically at their peak level of cognitive and neural profiles, leading to more uniform connectivity patterns across individuals. On the other hand, older adults seem to show more interindividual variability, reflecting a wider range of age-related changes in brain structure and function. This variability may be further shaped by differences in life experience such as level of mental engagement in day-to-day activities or differences in educational experiences. The age-related differences found on whole brain functional connectivity through the network and the RSA approach also support the observation regarding age-related dedifferentiation, such that older adults’ networks become less specialized (Betzel et al., 2014; Chan et al., 2014; King et al., 2018; Song et al., 2014; Spreng & Schacter, 2012), which has been related to declines in cognition (Chan et al., 2014).

Relating to language production, we found that resting state functional activity is significantly associated with speech performance, and importantly, these brain–behavior relationships differ by age group—highlighting the role of intrinsic brain activity in accounting for age-related differences in speech. With the univariate regression, we found that higher network segregation was associated with better speech performance in both the language network and the whole brain, and this relationship was primarily driven by younger adults. Older adults, on the other hand, did not show a significant association between network segregation and speech. This result is consistent with previous findings reported in Zhang and Diaz (2023), as well as other resting state (Antonenko et al., 2013) and task-based studies (Diaz et al., 2014) reporting weaker brain–behavior relationships among older adults. On the other hand, there was a significant positive relationship between activation intensity and speech performance across all participants, in both the language network and the whole brain. These results suggest that aging does not necessarily disrupt the contribution of local activation intensity to speech in the same way as it affects the large-scale network organization.

Yet, the univariate regression does not directly speak to how age-related differences in functional connectivity contribute to the differences in speech. RSA-based multivariate regression fills this gap by examining whether participants with dissimilar neural patterns also exhibit dissimilar behavioral profiles. Overall, the dissimilarity in language network as well as the whole brain positively related to dissimilarity in speech, suggesting that participants with dissimilar network structures had dissimilar speech performance. Importantly, the relationship between dissimilarity in resting state whole-brain activity—including both connectivity and amplitude of BOLD fluctuations—and dissimilarity in speech performance, was primarily driven by comparison between age groups (Figure 7D and F, and Figure 8B). This finding provides direct evidence that whole brain resting state activity plays a critical role in accounting for age-related differences in speech production.

Additionally, the RSA-based regressions suggest a dissociative pattern in the neural basis of speech production between age groups. As indicated in the integrated dissimilarity results (Figure 8), the brain-behavior relationship was strongest within younger adults for the language network, while stronger when capturing age group differences and individual differences within older adults for the whole brain network. When analyzing each brain dissimilarity dimension separately (Figure 7), although not all reached significance, the same trend occurred. Specifically, for language network (Figure 7A–C), younger adults (red lines) exhibit steeper slopes; whereas for the whole brain network (Figure 7D–F), between group comparison and within older adults (blue lines) show stronger brain–behavior relationships. These results suggest that for younger adults, the language network appears to be most critical, whereas for older adults, whole-brain activity—including both connectivity and spontaneous fluctuations—plays a more prominent role in accounting for individual differences in speech production. This pattern is consistent with compensatory recruitment accounts of cognitive aging, such as the compensation-related utilization of neural circuits hypothesis (CRUNCH), which propose that older adults engage additional neural resources to maintain performance (Reuter-Lorenz & Cappell, 2008). It also aligns with broader notions of domain-general scaffolding, where other brain systems are brought in to help support language functions in aging (Reuter-Lorenz & Park, 2014, 2024; Wierenga et al., 2008). In sum, this age-related dissociation may be overlooked using traditional univariate regression approaches, underscoring the value of multivariate methods such as RSA for capturing distributed brain–behavior correspondences.

It is worth noting that the older adult group was more variable in chronological age which may have contributed to the observed individual differences in dissimilarity measures (as shown in Figure 4). However, because our main outcome measures—specifically, the representational dissimilarity matrices—are based on pair-wise comparisons between individuals, it is not straightforward to incorporate individual age into regression models. To further explore this issue, we conducted additional exploratory analyses by dividing the older adult group into younger-old and older-old subgroups using a median split. Interestingly, the younger-old subgroup showed greater within-group whole-brain dissimilarity than the older-old subgroup (*t* = 3.16, *p* = 0.002), suggesting higher neural heterogeneity among the relatively younger subset of older adults. However, when examining how resting state dissimilarity contributed to speech dissimilarity within and between these subgroups, we found that the strongest brain–behavior relationships emerged in comparisons involving the older-old subgroup (*β* = 0.63, *t* = 2.69, *p* = 0.01) and in between-subgroup comparisons (*β* = 0.28, *t* = 3.20, *p* = 0.002). This pattern suggests that increased heterogeneity alone does not account for the observed effects. Rather, the findings point to an enhanced reliance on whole brain functional organization for speech performance as age advances within the older adult group. These results provide additional support for our interpretation that age-related brain–behavior dynamics shift over the course of late adulthood.

### Limitations

Although informative, the current study has several limitations. First, while our exploratory factor analysis aimed to capture a shared construct of speech quality and the factor loadings on the variables were very high (≥0.63; Costello & Osborne, 2005; Hair et al., 2009), the model fit was suboptimal (e.g., root-mean-square error of approximation = 0.27, Tucker-Lewis index = 0.65), suggesting that fluency and complexity may not be fully unified under a single factor. Thus, while the composite measure provides a useful summary, future studies may consider examining individual speech dimensions (e.g., complexity, vocabulary, disfluency) separately to further refine our understanding of their neural correlates. Second, while the study revealed reliable age-related differences in brain–behavior relationships, the relatively small sample size in the older adult group may limit generalizability. Although nonparametric permutation tests further confirmed the robustness of all regression analyses, future work should replicate these results in larger samples. Furthermore, the current study had unbalanced gender distribution, with a predominance of female participants—particularly among older adults. Future studies should aim for more gender-balanced samples to better examine potential sex-related effects. Finally, although resting state dynamics may be related to both stable individual traits and transient state related influences, our use of multiple complementary measures—including connectivity, network organization, and signal intensity—helps provide a more robust neural profile. Nevertheless, future work should combine resting and task-based imaging to better disentangle trait and state effects.

### Conclusion

Older adults exhibited significantly poorer speech and less efficient resting state brain activity, reflected by less segregated whole brain networks, lower ALFF, and more heterogeneous brain states across individuals. Despite these changes in the whole brain, the language network remains largely stable with age. Additionally, resting state brain states closely relate to speech performance. Univariate regression assessed how individual brain features directly influence speech, showing that younger adults might show stronger relationships between specific network features and speech performance due to more robust or efficient neural networks, whereas older adults might have more variability that weakened these direct relationships. Although univariate regression can show if brain–behavior relationships vary by age, it does not explain how the differences in brain connectivity relate to age-related behavioral differences. Multivariate RSA-based regression fills this gap by showing that the dissimilarity in resting state brain states is critically related to age-related differences in speech production. Furthermore, speech production in younger adults relies primarily on the language network, whereas in older adults it depends more on the overall functional state of the whole brain. Overall, the results from the current study suggest that outside of the language network, the whole brain resting state profile could be a valuable stable biomarker of age-related differences in speech production. While not yet clinically actionable, such measures offer a quantifiable and noninvasive index of brain–behavior coupling that may, in future longitudinal work, help track cognitive-speech decline or differentiate healthy from pathological aging trajectories.

## ACKNOWLEDGMENTS

We thank members of the Language Aging and Bilingualism Lab for their help with data collection. We thank the staff at the Centre for Cognitive and Brain Sciences (CCBS) at the University of Macau, where the study was conducted, for their support.

## FUNDING INFORMATION

Haoyun Zhang, National Natural Science Foundation of China (https://dx.doi.org/10.13039/501100001809), Award ID: 32200845. Haoyun Zhang, The Science and Technology Development Fund, Macao SAR, Award ID: FDCT, 0153/2022/A.

## AUTHOR CONTRIBUTIONS

**Haoyun Zhang**: Conceptualization: Lead; Formal analysis: Equal; Funding acquisition: Lead; Methodology: Lead; Project administration: Lead; Supervision: Lead; Visualization: Equal; Writing – original draft: Lead; Writing – review & editing: Equal. **Keikei Lei**: Conceptualization: Supporting; Formal analysis: Supporting; Visualization: Equal; Writing – review & editing: Equal. **Hanxiang Yu**: Data curation: Supporting; Formal analysis: Supporting. **Megan Nakamura**: Conceptualization: Supporting; Writing – review & editing: Supporting. **Michele Diaz**: Writing – review & editing: Supporting.

## DATA AND CODE AVAILABILITY STATEMENT

Data and analysis scripts can be found at: https://osf.io/7f695/.

## Supplementary Material



## TECHNICAL TERMS

Network segregation: The degree to which brain networks operate independently from each other.
Amplitude of low-frequency fluctuations (ALFF): A measure of spontaneous brain activity based on low-frequency signal changes.
Representative similarity analysis (RSA): A multivariate technique that compares similarity across individuals based on patterns of brain activity or behavioral performance.
Resting state functional connectivity: Measures how different brain regions activate and interact when a person is not engaged in any specific task.
